# Perceived family functioning and its association with depressive symptoms severity and quality of life in patients with major depressive disorder

**DOI:** 10.1192/j.eurpsy.2023.520

**Published:** 2023-07-19

**Authors:** K. Koutra, G. Mavroeides, M. Basta, A. Vgontzas

**Affiliations:** ^1^Department of Psychology, University of Crete, Rethymnon; ^2^Department of Psychiatry & Behavioral Sciences, Faculty of Medicine; ^3^Mobile Mental Health Unit, University Hospital of Heraklion, University of Crete, Heraklion, Greece; ^4^Sleep Research & Treatment Center, Department of Psychiatry & Behavioral Health, Penn State College of Medicine, Hershey, PA, United States

## Abstract

**Introduction:**

Studies have shown that family factors affect the development, maintenance and course of major depressive disorder (MDD).

**Objectives:**

The present study aimed to prospectively investigate whether dysfunctional family functioning is associated with meaningful clinical outcomes including symptom severity and quality of life (QoL) in patients with MDD.

**Methods:**

A total of 114 patients with a clinical diagnosis of MDD (83.3% females, aged 47.25±13.98 years) participated in the study. Participants were recruited from the outpatient clinic, Department of Psychiatry and the mobile mental health unit of the University Hospital of Heraklion in Crete, Greece, and from a Greek online depression peer-support group. Family functioning was assessed in terms of cohesion, flexibility, communication and satisfaction dimensions (FACES IV) at baseline. Depression severity (BDI) and QoL (WHOQOL-BREF) were assessed about 10 months after the baseline assessment (9.56±2.52).

**Results:**

Conceptually, the cohesion dimension contains Balanced Cohesion (central area) with Disengaged (low unbalanced) and Enmeshed (high unbalanced) dimension, and the flexibility dimension contains Balanced Flexibility (central area) with Rigid (low unbalanced) and Chaotic (high unbalanced) dimension. Multivariable analysis adjusting for confounding variables such as patients’ educational level, residence, family structure, pharmacotherapy, psychotherapy, and history of suicide attempts indicated that Balanced Cohesion was positively associated with increased levels of patients’ psychological QoL. Moreover, two out of four unbalanced scales - Enmeshed and Chaotic - were negatively related to lower psychological QoL. The findings also demonstrated that Enmeshed scale was positively associated with higher depressive symptoms. Finally, lower family communication was related to increased depressive symptoms, whereas lower family satisfaction was associated with patients’ lower psychological QoL.

**Conclusions:**

Family environmental factors appear to play an important role in clinical outcomes of MDD. Family interventions targeting dysfunctional family interactions by promoting awareness of family dynamics could improve the emotional well-being of patients with MDD.

**Disclosure of Interest:**

None Declared

